# Assessment of the reporting quality of randomised controlled trials of massage

**DOI:** 10.1186/s13020-021-00475-6

**Published:** 2021-07-28

**Authors:** Xuan Zhang, Lin Zhang, Weifeng Xiong, Xihong Wang, Xiaohan Zhou, Chen Zhao, Guihua Tian, Hongcai Shang, Taixiang Wu, Jiangxia Miao, Zhaoxiang Bian

**Affiliations:** 1 Chinese Clinical Trial Registry (Hong Kong), Hong Kong Chinese Medicine Clinical Study Centre, School of Chinese Medicine, Hong Kong Baptist University, 307 Room, Jockey Club School of Chinese Medicine Building, 7 Baptist University Road, Kowloon Tong, Kowloon, Hong Kong, HKSAR China; 2 Chinese EQUATOR Centre, Hong Kong Baptist University, Hong Kong, HKSAR China; 3 Tianjin University of Traditional Chinese Medicine, Tianjin, China; 4 College of Chinese Medicine, Beijing University of Chinese Medicine, Beijing, China; 5 Institute of Basic Research in Clinical Medicine, China Academy of Chinese Medical Sciences, Beijing, China; 6 Key Laboratory of Chinese Internal Medicine of Ministry of Education and Beijing, Dongzhimen Hospital, Beijing University of Chinese Medicine, Beijing, China; 7 Chinese Cochrane Centre, West China Hospital, Sichuan University, China Trial Registration Center, Chengdu, Sichuan China; 8 School of Chinese medicine, The Chinese University of Hong Kong, Hong Kong, HKSAR China

**Keywords:** CONSORT Extension, Tuina, Massage, Nonpharmacologic treatment, Randomised controlled trials (RCTs), Reporting quality

## Abstract

**Objective:**

To assess the reporting quality of randomised controlled trials (RCTs) of massage, particularly whether necessary elements related to massage interventions were adequately reported.

**Methods:**

A total of 8 electronic databases were systematically searched for massage RCTs published in English and Chinese from the date of their inception to June 22, 2020. Quality assessment was performed using three instruments, namely the CONSORT (Consolidated Standards of Reporting Trials) 2010 Checklist (37 items), the CONSORT Extension for NPT (Nonpharmacologic Treatments) 2017 checklist (18 items), and a self-designed massage-specific checklist (16 items) which included massage rationale, intervention and control group details. Descriptive statistics were additionally used to analyse the baseline characteristics of included trials.

**Results:**

A total of 2,447 massage RCTs were identified, of which most (96.8%) were distributed in China. For the completeness of CONSORT, NPT Extension, and massage-specific checklists, the average reporting percentages were 50%, 10% and 45%, respectively. Of 68 assessed items in total (exclusion of 3 repeated items on intervention), 42 were poorly presented, including 18 CONSORT items, 15 NPT items, and 9 massage-specific items. Although the overall quality of reporting showed slightly improvement in articles published after 2010, the international (English) journals presented a higher score of the CONSORT and NPT items, while the Chinese journals were associated with the increased score of massage-specific items.

**Conclusion:**

The quality of reporting of published massage RCTs is variable and in need of improvement. Reporting guideline “CONSORT extension for massage” should be developed.

**Supplementary Information:**

The online version contains supplementary material available at 10.1186/s13020-021-00475-6.

## Background

Massage therapy (MT), an ancient form of medical technique, can be defined as a systematic manipulation of soft tissues of the body for pain reduction or other therapeutic purposes [1]. Archaeological studies have unearthed evidence of massage dating back to around 2700 BC, making it the forerunner of all other forms of massage and body work that exist today, from shiatsu to osteopathy [2]. Indeed, massage seems to have been the primary form of healing throughout history in places such as ancient Greece, where Hippocrates (the father of modern medicine) described medicine as “being the art of rubbing” [3]. In China, the earliest record of massage was in the Shang dynasty (c. 1600 BC), when it was called “Anmo” (e.g., pressing and rubbing). The most popular term, however, for Chinese massage is “Tuina” (e.g., pushing and grasping), a term that appeared in the first specialised book of pediatric tuina published in the Ming dynasty (c. 1400) [4]. As a traditional intervention of Chinese medicine (CM), a wide range of technical manipulations, such as pulling, pressing, tapping, shaking, vibrating, kneading, rolling, gliding, and acupressure, are included in Tuina therapy for stimulating meridians, relaxing muscles, relieving pain and stretching joints [5]. Besides CM, there are different styles in other countries, such as Swedish massage, Thai massage, oil massage, and aromatherapy massage [6]. With the functions of boosting blood circulation, removing blockages, realigning the musculoskeletal structure, and enhancing the body’s natural healing abilities, massage practice has been, and continues to be, a vital part of complementary and alternative medicine (CAM) treatments around the world [7].

Recently, there is an increasing number of clinical trials on massage. These studies suggested that MT has been notably effective in enhancing the growth of infants, increasing attentiveness, decreasing depression and aggression, alleviating motor problems, reducing pain, and enhancing immune function [8]. However, some scholars have indicated that the quality of available massage trials is not optimal due to flaws in reporting and methodology [9]. Incomplete reporting is one such shortcoming, which not only affects the efficacy evaluation of massage interventions, but also compromises the grades of research evidence, even misleading the clinical practice [10]. Generally, “reporting quality” is the basis for both peer reviewers’ and readers’ judgments of scientific merit, of truth, of accuracy, and of clinical applicability. However, “reporting quality” is a general term that needs to be translated into specific criteria covering study rationale, trial design, measurements and analysis. Massage trials involve aspects that do not present in other types of trials or studies. Specifically, for massage trials, details of the massage environment, patients’ posture, media used for massage, the locations, force, frequency, and duration of massage, manipulations and procedures, treatment provider background, etc., are critical for trial assessment and replication. Although the Consolidated Standards of Reporting Trials (CONSORT) Statement and its extension for nonpharmacological treatments (NPT) provide researchers with clear reporting checklists for RCTs [11, 12], no previous study has assessed whether massage-specific details are sufficiently reported in the current massage RCTs; nor has any study identified what key information affecting the quality of massage trials should be described in the reports.

Therefore, in this review, the assessments of reporting quality of massage trials were not only based on the checklists of CONSORT 2010 and NPT Extension 2017, but also on a self-designed massage-specific checklist (e.g., the rationale of massage selected, details of manipulation and techniques, description of the treatment provider and control groups). The objectives of this study were as follows: (a) to summarize the general characteristics of published RCTs on massage; (b) to assess their reporting quality based on the standard CONSORT and NPT checklists; and (c) to evaluate whether necessary information related to massage interventions were adequately reported according to the massage-specific items.

## Methods

### Inclusion and exclusion criteria

This study included RCTs with massage intervention(s) published in English and Chinese up to 22 June 2020. We included a wide range of massage therapy, such as Chinese massage (Tuina), Swedish massage, Thai massage, Malay massage, ice massage, oil massage, infant massage, abdomen massage, and foot massage. Massage intervention(s) may have been administered alone or in combination with other interventions of conventional Western medicine or CAM. There were no limitations in the types of participants and controls in the included studies. Repeat publications, non-randomised or non-controlled trials, non-massage interventional trials, comprehensive interventions not focused on massage treatment, study protocols, reviews, observational studies, case reports, abstracts or full-text reports not found, non-human studies, and non-English or non-Chinese language reports were excluded.

### Search strategy

The following eight databases, EMBASE, MEDLINE, Evidence-Based Medicine (EBM) Reviews, Allied and Complementary Medicine (AMED), The China National Knowledge Infrastructure (CNKI), VIP database, Wanfang database, and WHO International Clinical Trials Registry Platform (including 17 registries updated in June 2020), were searched from the date of their inception to June 22, 2020. Languages were restricted to English and Chinese. The search terms were “massage”, “Chinese medicine”, “manipulative”, “reflexion therapy”, “physical contact”, “randomised”, “randomisation”, “randomized”, and “clinical trial”, etc. The detailed search strategy is given in Additional file 1: Appendix S1.

### Screening

The titles and abstracts of the records were independently screened by two authors (LZ and WFX) based on selection criteria, and the full texts of potentially suitable articles were retrieved for further assessment. Disagreements, if any, were resolved by discussion or consultation with the third author (XZ).

### Data extraction

Two authors (LZ and XZ) independently extracted details on a number of general characteristics of included studies, mainly involving publication years and journals, authors’ information, type of studied diseases, interventions and outcomes, and study design (e.g., assignment, blinding and sample size). To ensure consistency in the data extraction process, criteria for the extraction items and typical examples were developed and used on twenty articles (e.g., ten English papers and ten Chinese papers) for testing, and then subsequently used to extract data from all included records. When there was any uncertainty regarding a particular article, the data extracted were checked and resolved by the third researcher (ZXB).

### Assessment of reporting quality

The reporting quality of included trials was evaluated according to (1) the 37-item (including sub-items) of the CONSORT 2010 checklist; (2) the 18-item of the CONSORT Extension for Trials of Nonpharmacologic Treatments (NPTs) 2017 checklist; and (3) the 16-item of a self-designed massage-specific checklist. This massage-related checklist was developed by three researchers (ZXB, GHT, and XZ) based on a discussion about (1) the key elements of massage in clinical practice; (2) transparent reporting of massage details in the clinical trials, such as the treatment posture, media used, location selected, manipulations and procedures, patients’ responses, and adverse effects; (3) the rationale of why massage intervention(s) was selected; and (4) relevant items from the CONSORT Extensions for Chinese herbal medicine (CHM) formula, acupuncture, moxibustion, and cupping [13–16]. Aiming for easy calculation, the specifics of this checklist were categorized into six items with sixteen sub-questions, covering massage rationale, details of technique, treatment regimen, other components of treatment, massage provider background, and control or comparator of massage.

There is a total of 71 items (e.g., CONSORT 37-item, NPT 18-item, and massage-specific 16-item) in the above evaluation tools. As the massage-specific checklist was focused on an extension for the details of intervention, we thereby did not assess the three generalised items in the CONSORT and NPT Extension which with the same contents of the intervention, namely item 5 intervention of the CONSORT, and item 5a and 5b of NPT Extension. Therefore, a total of 68 items (including CONSORT 36-item, NPT 16-item, and massage-specific 16-item) were actually assessed in this study. Each item/question was scored as ‘‘yes’’ if it was fully reported (Y, marked as 1 point) or ‘‘no’’ if it was incompletely reported or absent (N, marked as 0 point). The details of scoring rules are presented in Additional file 1: Appendix S2. Each included article was assessed by two researchers (LZ and XZ) independently, and the results were double-checked. Any problems or ambiguities that arose were resolved by the third researcher (ZXB).

### Statistical analysis

For each assessed item, the number (n) and percentage (%) of trials that completely reported the item were provided. The reporting percentages were arbitrarily categorized as excellent (>85%), good (between 50% and 85%), and poor (< 50%). The mean overall CONSORT and NPT scores (e.g., the full score is 52), and massage-specific scores (e.g., the full score is 16) were calculated by subgroups and recorded as mean and standard deviations (SD). As indicated in the previous studies, the development of reporting guideline is closely related to the reporting quality of clinical trials [17]. Some scholars have identified that the publish year of guideline and types of journals (e.g., various in the reporting requirements and endorsement degree of the guideline) are the most essential and direct factors related to reporting quality [18, 19]. Therefore, the selection of subgroups focused on (1) Year group, and (2) Journal group. Further, as the CONSORT 2010 guideline is the standard reporting framework for the RCTs, the Year group was separated as before (1990-2010) and after (2011-2020) the year of 2010. Regarding different types of journals, the major difference of reporting requirements is commonly existing between Chinese journals and international journals [20, 21]. Based on the language selection in this study, the Journal group was defined as the “Chinese journal” and the “International (English) journal”. All data were collected and recorded in Microsoft Office Excel (Version 2016). Descriptive statistics were performed using SPSS software, version 25.0.

## Results

### Search

**Fig. 1:**
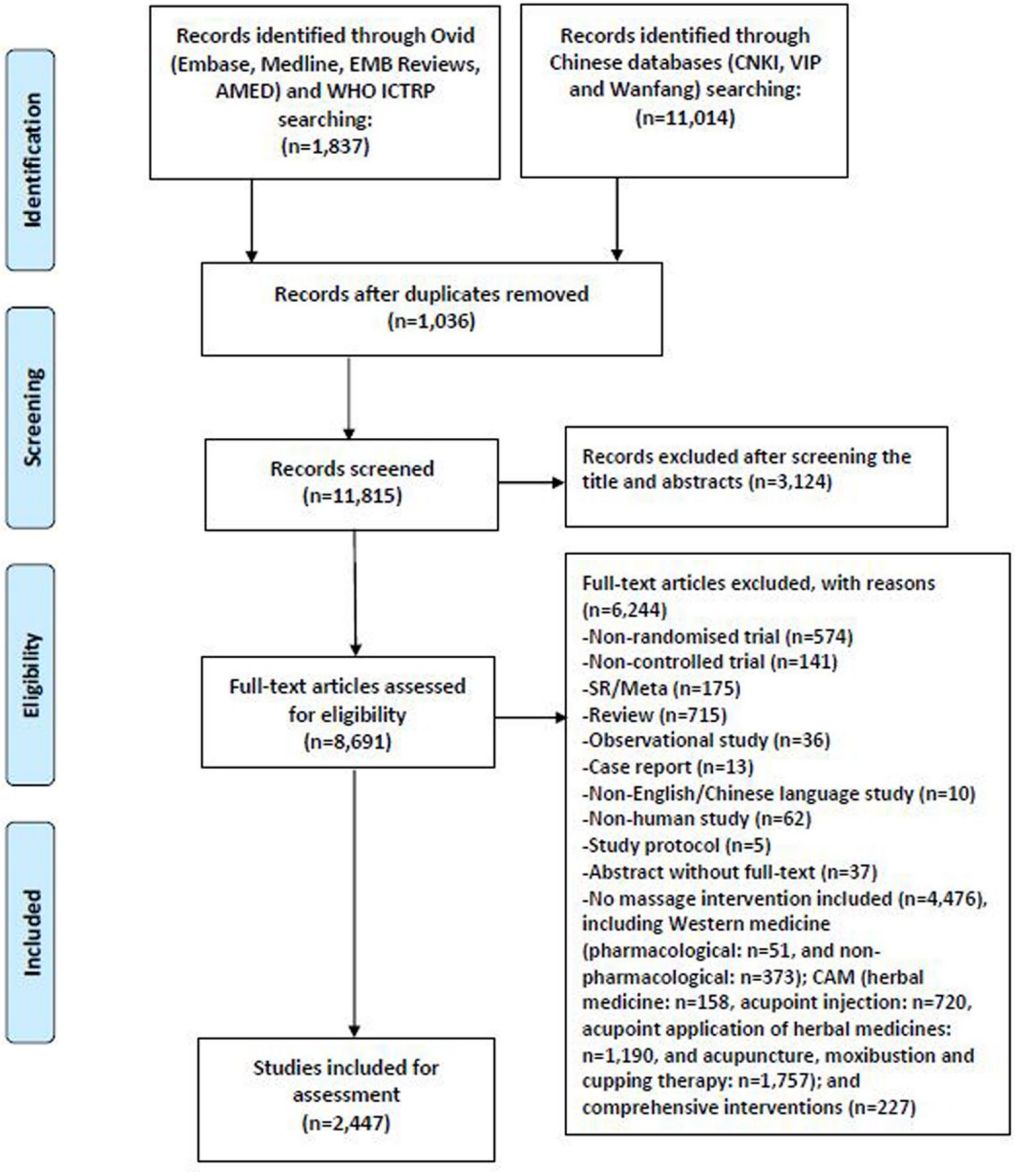
Flow chart of the search and selection process

The initial search identified a total of 12,851 records, of which 8,691 relevant studies were retained after excluding duplicates and screening the titles and abstracts. After reviewing the full text of these articles, we identified 2,447 RCTs of massage for inclusion in our final analysis (Fig. 1).

### Characteristics of included trials

**Fig. 2:**
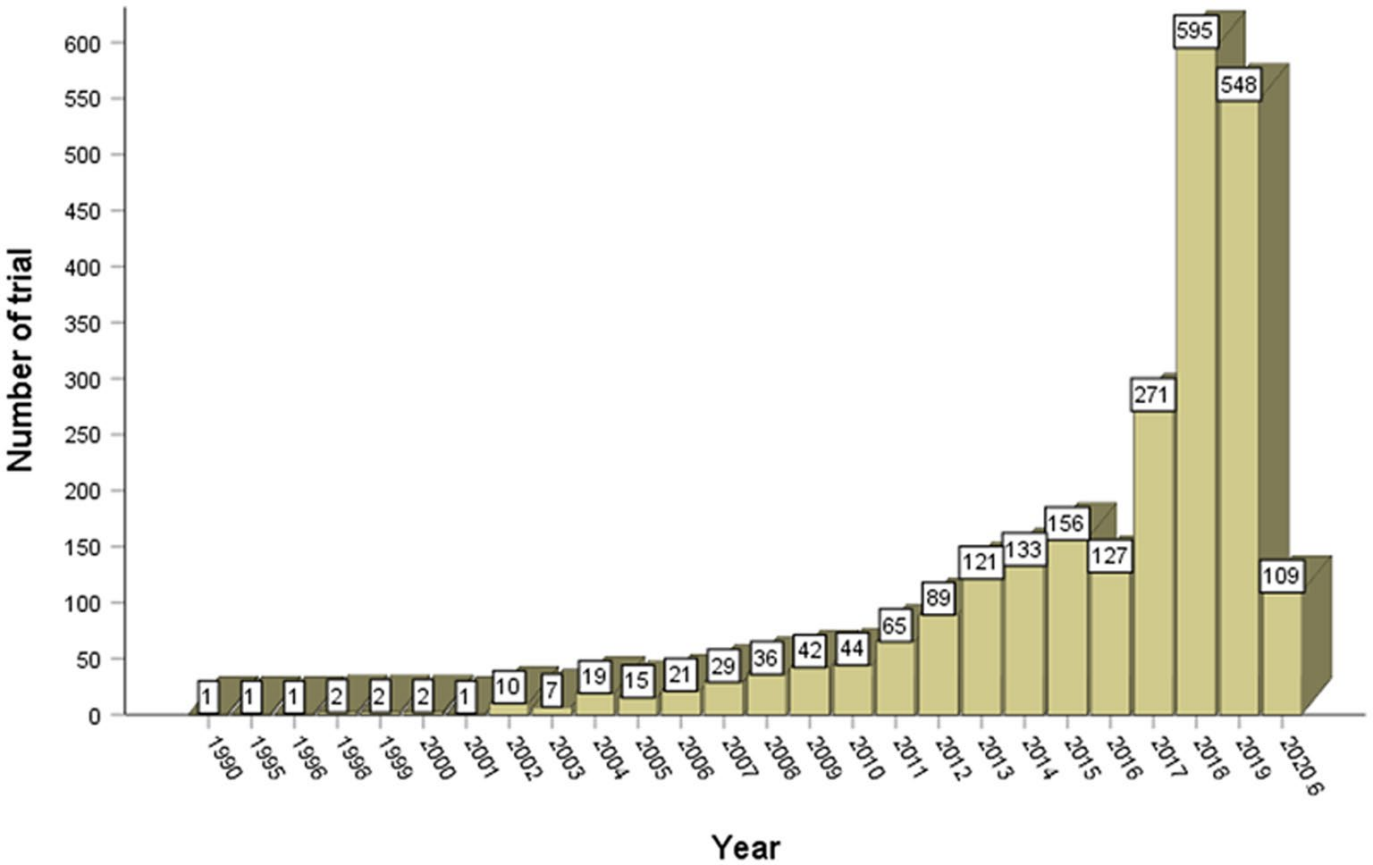
Number of massage RCTs published from 1990 to June 2020

**Fig. 3:**
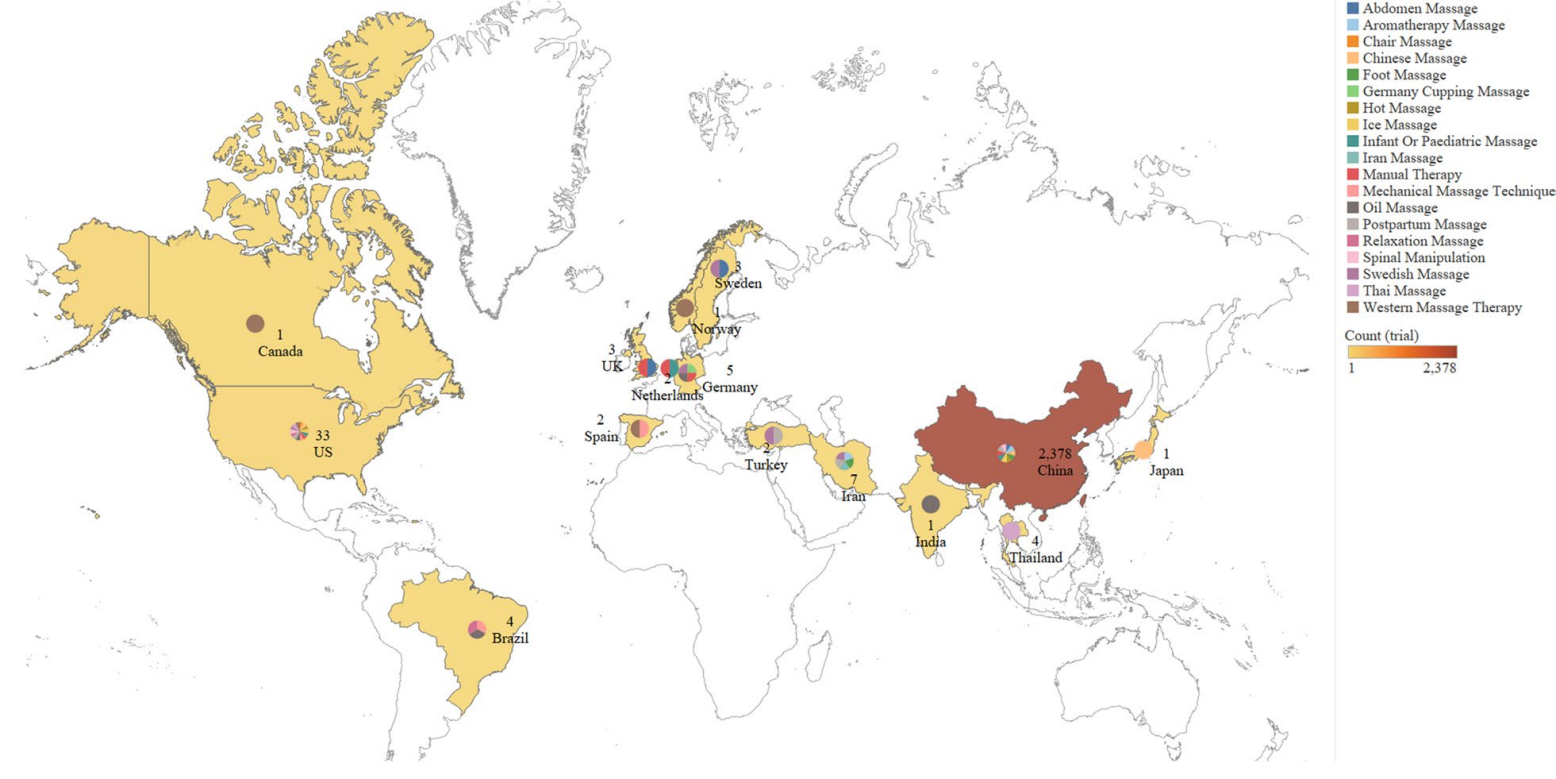
The geographic distribution and massage types of included trials

The earliest RCT of massage included in this study was published in 1990. Since 2011, the number of trials has increased markedly, and more than half (57.8%) were published during 2017–2019 (Fig. 2). All 2,447 trials were distributed in 15 countries, of which China makes the greatest contribution to the number of massage RCTs. Among 19 reported types of massage interventions, Chinese massage (also called Tuina which is based on the CM theory) was identified as the most common style (Fig. 3).

Most of the included trials (96.8%) were published in Chinese journals, of these journals, only 29.6% were classified as medical core journals. The number of authors was mainly 1–5 (87.5%), but 36.8% of articles reported only one author. The most common condition studied is the disease of musculoskeletal system or connective tissue (44.1%). Commonly, massage RCTs were performed in a single centre (97.6%), with a sample size of 51–100 (56.5%), and with two assignment groups (95.7%). Of 2,447 trials, 45.3% did not obtain or provide ethical approval, and 23.5% did not declare a conflict of interest. Few trials used the blinding methods (3.2%) for massage interventions, and less than 15% of trials adopted the CM pattern (or other CM-related indicators) in the assessed conditions and outcomes. 72.1% of articles have been cited less than five times (Table 1).

**Table 1.** Characteristics of included articles (n = 2447)

Characteristic	*n* (%)
Languages of published journals	
Chinese	2368 (96.8)
English	79 (3.2)
Type of published journals	
Comprehensive medical journals	936 (38.3)
CAM/CM specific journals	1288 (52.6)
Massage specific journals	223 (9.1)
Grades of Chinese journals	
Medical core journals^a^ (Yes)	702 (29.6)
Impact factor of international (English) journals^b^	
0–4	75 (94.9)
5–10	1 (1.3)
> 10	3 (3.8)
Number of authors included	
1	901 (36.8)
2–5	1241 (50.7)
6–10	289 (11.8)
> 10	16 (0.7)
Institution of the corresponding author	
Hospital	2279 (93.1)
University	137 (5.6)
Research Institute	28 (1.1)
Others	3 (0.1)
Type of conditions treated (top five)	
Diseases of the musculoskeletal system or connective tissue	1,078 (44.1)
Symptoms, signs or clinical findings, not elsewhere classified	249 (10.2)
Diseases of the nervous system	236 (9.6)
Pregnancy, childbirth or the puerperium	180 (7.4)
Diseases of the digestive system	174 (7.1)
Studied disease(s) with CM Pattern(s)	
Yes	155 (6.3)
Experimental group	
Only massage intervention(s)	664 (27.1)
Other interventions administered to massage	1783 (72.9)
Type of blinding	
Single	60 (2.5)
Double	16 (0.7)
Open label	2,306 (94.2)
Not reported	65 (2.7)
Number of assigned groups	
2	2342 (95.7)
3	80 (3.3)
> 3	25 (1.0)
Sample size	
1–50	239 (9.8)
51–100	1383 (56.5)
101–300	764 (31.2)
301–500	38 (1.6)
> 500	23 (1.0)
Trial participating centre	
Single centre	2388 (97.6)
Multi-centre	59 (2.4)
Outcomes including CM-related indicators	
Yes	181 (7.4)
Number of times cited	
0	566 (23.1)
1–5	1200 (49.0)
6–10	339 (13.9)
11–20	229 (9.4)
> 20	113 (4.6)
Ethics approval	
Yes	1339 (54.7)
Declaration of conflict of interest	
Yes	1871 (76.5)

*CAM* complementary and alternative medicine, *CM* Chinese medicine

^a^According to the 2019 China Science and Technology Core Journals Catalog (Natural Science Volume) [22]

^b^The impact factor (IF) of an academic international journal is a scientometric index calculated by Clarivate that reflects the yearly average number of citations of articles published in the last two years in a given journal. The record of IF was collected from the journal formal website of each targeted journal in Oct 2020.

### Reporting quality of included trials

As for completeness in reporting, i.e., the number of checklist items reported in a given RCT, the average reporting rates were 50% (0.2–99.8%) for CONSORT, 10% (0.04–57%) for NPT Extension, and 45% (1.8–94.4%) for massage-specific items, respectively. For all 68 assessed items, reporting quality of 29% (20 items) was “excellent” (> 85%) and of 9% (6 items) was “good” (50 85%); but 62% (42 items) were poorly reported (< 50%). Specifically, the poor reporting referred to 18 CONSORT items, 15 NPT items, and 9 massage-specific items. Details are provided in Tables 2 and 3.

**Table 2.** Reporting quality of 2,447 included trials based on the CONSORT and CONSORT for NPT items

Section/topic	Item number and description	Item present, n (%)
Title and abstract	1a. Identification as a randomised trial in the title	656 (26.8)
	1b. Structured summary of trial design, methods, results, and conclusions (for specific guidance see CONSORT for abstracts)	2420 (98.9)
	*1b NPT. When applicable, report eligibility criteria for centers where the intervention is performed and for care providers*	1395 (57.0)
	*1b NPT. Report any important changes to the intervention delivered from what was planned*	543 (22.2)
Introduction		
Background and objectives	2a. Scientific background and explanation of rationale	2364 (96.7)
	2b. Specific objectives or hypotheses	2395 (97.9)
Methods		
Trial design	3a. Description of trial design (such as parallel, factorial) including allocation ratio	77 (3.1)
	*3a NPT. When applicable, how care providers were allocated to each trial group*	945 (38.6)
	3b. Important changes to methods after trial commencement (such as eligibility criteria), with reasons	1676 (68.5)
Participants	4a. Eligibility criteria for participants	2400 (98.1)
	*4a NPT. When applicable, eligibility criteria for centers and for care providers*	52 (2.1)
	4b. Settings and locations where the data were collected	2431 (99.3)
Interventions	5. Interventions for each group with sufficient details to allow replication, including how and when they were actually administered	See Table 3
	*5a NPT. Precise details of both the experimental treatment and comparator*	See Table 3
	*5b NPT. Description of the different components of the interventions and, when applicable, description of the procedure for tailoring the interventions to individual participants.*	See Table 3
	*5c NPT. Details of whether and how the interventions were standardized.*	943 (38.5)
	*5d NPT. Details of whether and how adherence of care providers to the protocol was assessed or enhanced*	121 (4.9)
	*5e NPT. Details of whether and how adherence of participants to interventions was assessed or enhanced*	7 (0.3)
Outcomes	6a. Completely defined pre-specified primary and secondary outcome measures, including how and when they were assessed	1431 (58.5)
	6b. Any changes to trial outcomes after the trial commenced, with reasons	4 (0.2)
Sample size	7a. How sample size was determined	844 (34.5)
	*7a NPT. When applicable, details of whether and how the clustering by care providers or centers was addressed*	3 (0.1)
	7b. When applicable, explanation of any interim analyses and stopping guidelines	10 (0.4)
Sequence generation	8a. Method used to generate the random allocation sequence	652 (26.6)
	8b. Type of randomisation; details of any restriction (such as blocking and block size)	78 (3.2)
Allocation concealment mechanism	9. Mechanism used to implement the random allocation sequence (such as sequentially numbered containers), describing any steps taken to conceal the sequence until interventions were assigned	74 (3.0)
Implementation	10. Who generated the random allocation sequence, who enrolled participants, and who assigned participants to interventions	46 (1.9)
Blinding	11a. If done, who was blinded after assignment to interventions (for example, participants, care providers, those assessing outcomes) and how	2343 (95.7)
	*11a NPT. If done, who was blinded after assignment to interventions (e.g., participants, care providers, those administering co-interventions, those assessing outcomes) and how*	27 (1.1)
	11b. If relevant, description of the similarity of interventions	23 (0.9)
	*11c NPT. If blinding was not possible, description of any attempts to limit bias*	12 (0.5)
Statistical methods	12a. Statistical methods used to compare groups for primary and secondary outcomes	2373 (97.0)
	*12a NPT. When applicable, details of whether and how the clustering by care providers or centers was addressed*	1 (0.04)
	12b. Methods for additional analyses, such as subgroup analyses and adjusted analyses	10 (0.4)
Results		
Participant flow (a diagram is strongly recommended)	13a. For each group, the numbers of participants who were randomly assigned, received intended treatment, and were analysed for the primary outcome	2440 (99.7)
	*13a NPT. The number of care providers or centers performing the intervention in each group and the number of patients treated by each care provider or in each center*	3 (0.1)
	13b. For each group, losses and exclusions after randomisation, together with reasons	2441 (99.8)
	*13c NPT. For each group, the delay between randomization and the initiation of the intervention*	2 (0.1)
Recruitment	14a. Dates defining the periods of recruitment and follow-up	2376 (97.1)
	14b. Why the trial ended or was stopped	2367 (96.7)
Baseline data	15. A table showing baseline demographic and clinical characteristics for each group	324 (13.2)
	*15 NPT. When applicable, a description of care providers (case volume, qualification, expertise, etc.) and centers (volume) in each group*	4 (0.2)
Numbers analysed	16. For each group, number of participants (denominator) included in each analysis and whether the analysis was by original assigned groups	2336 (95.5)
Outcomes and estimation	17a. For each primary and secondary outcome, results for each group, and the estimated effect size and its precision (such as 95% confidence interval)	2319 (94.8)
	17b. For binary outcomes, presentation of both absolute and relative effect sizes is recommended	2183 (89.2)
Ancillary analyses	18. Results of any other analyses performed, including subgroup analyses and adjusted analyses, distinguishing pre-specified from exploratory	8 (0.3)
Harms	19. All important harms or unintended effects in each group (for specific guidance see CONSORT for harms)	148 (6.0)
Discussion		
Limitations	20. Trial limitations, addressing sources of potential bias, imprecision, and, if relevant, multiplicity of analyses	117 (4.8)
	*20 NPT. In addition, take into account the choice of the comparator, lack of or partial blinding, and unequal expertise of care providers or centers in each group*	10 (0.4)
Generalisability	21. Generalisability (external validity, applicability) of the trial findings	2185 (89.3)
	*21 NPT. Generalizability (external validity) of the trial findings according to the intervention, comparators, patients, and care providers and centers involved in the trial*	3 (0.1)
Interpretation	22. Interpretation consistent with results, balancing benefits and harms, and considering other relevant evidence	2308 (94.3)
Other information		
Registration	23. Registration number and name of trial registry	57 (2.3)
Protocol	24. Where the full trial protocol can be accessed, if available	38 (1.6)
Funding	25. Sources of funding and other support (such as supply of drugs), role of funders	495 (20.2)

**Table 3.** Assessment on the self-designed massage-specific checklist

Item	Specifics	Yes, n (%)
Massage rationale	Q1. Whether the type of massage was reported?	1504 (61.5)
	Q2. Whether the rationale for selected massage was provided?	975 (39.8)
	Q3. Whether any information about individualized massage treatment was reported?	82 (3.4)
Details of massage	Q4. Whether the patient posture and/or environment during treatment was mentioned?	1697 (69.4)
	Q5. Whether the media (e.g., dosage and manufacturers) used for massage was reported?	351 (14.3)
	Q6. Whether the massage points and/or locations were provided?	2311 (94.4)
	Q7. Whether the duration, frequency and/or force of massage in per point and/or location was reported?	2088 (85.3)
	Q8. Whether the details of procedure and technique of massage was described?	1877 (76.7)
	Q9. Whether any responses sought of patients during/after massage was reported?	690 (28.2)
	Q10. Whether any measures or management for possible adverse events was pre-mentioned?	45 (1.8)
Treatment regimen	Q11. Whether the number, frequency, duration and/or force of provided massage sessions was reported?	2297 (93.9)
Other components of treatment	Q12. For complex interventions, whether the details of other interventions administered to the massage group were reported?	2159 (88.2)
	Q13. Whether any instruction and/or information of selected massage was presented to treatment providers and the participants?	83 (3.4)
Treatment provider background	Q14. Whether any description of treatment providers’ background (e.g., qualification and/or experiences in massage) was reported?	149 (6.1)
Control or comparator interventions	Q15. Whether the rationale for the choice of the control(s) was provided?	73 (3.0)
	Q16. Whether the details of control or comparator were described?	1214 (49.6)

In the subgroup of before and after the year of 2010, the overall reporting scores increased slightly for both CONSORT and NRT items, and massage-specific items from 19.6 (2.9) to 19.8 (2.8), out of 52 in total, and from 7.3 (1.9) to 7.6 (1.9), out of 16 in total. In the comparison of journals, the international (English) group showed a higher score of 22.7 (3.7) in the CONSORT and NRT items, and the Chinese journals associated with the increased score of 7.6 (1.9) in the massage-specific items (Table 4).

**Table 4.** Overall reporting quality scores for 2,447 included trials, by subgroup

Subgroup	For massage-specific items	
	Mean (SD) score^a^	Mean (SD) score^b^
Year of publication (*n*)		
1990–2010 (*n*=233)	19.6 (2.9)	7.3 (1.9)
2011–2020 (*n*=2,214)	19.8 (2.8)	7.6 (1.9)
Journal of publication (n)		
Chinese journal (*n *= 2368)	20.1 (2.6)	7.6 (1.9)
International (English) journal (*n *= 79)	22.7 (3.7)	5.5 (1.5)

*SD* standard deviation

^a^A perfect score is 52 for the CONSORT and NPT items

^b^A perfect score is 16 for the massage-specific items

## Discussion

This study provides a literature review of the reporting characteristics and quality of massage RCTs in the past 30 years, which have identified a remarkable increase in the number of publications since 2017. Although RCT is regarded as the gold standard among different types of clinical studies, the quality of massage RCTs included in this review appears to be far from optimal. Incomplete or absent reporting of key trial information leads to some difficulties for assessing the quality of the whole study, easily inviting skepticism as to its results. Similar problems were also found in the previous study examining systematic reviews of massage treatments [10].

In this study, we found several problems in the characteristics of articles reporting massage RCTs. First, few studies were published in relatively high-quality journals. For the 96.8% of trials published in Chinese journals, only 702 articles were published in the “Chinese Medical Core Journals” [22]. For the remaining 3.2% published in international (English) journals, only 4 articles were rated as having a relatively high-impact factor (e.g., IF > 5). Higher-quality journals usually have broader reviewer networks and more rigorous reporting requirements. Previous studies have indicated that the reporting quality of clinical trials is positively correlated with journal quality [19]. Secondly, CM-related indicators were rarely mentioned in the design of massage RCTs. Although CM-based massage intervention (e.g., Tuina/Chinese massage) was the most commonly used, less than 15% of trials adopted CM pattern as diagnostic criteria and outcome(s) for evaluation. Previous studies have pointed out that if a CM trial does not consider CM pattern identification (‘zheng’) or use CM ‘zheng’-related outcome(s), participants may not be properly treated and/or the efficacy of CM intervention(s) may not be assessed properly [23]. Thirdly, the common study design was a two-arm-in-parallel group, with 51-100 sample size, open label and a single centre trial. Thus, high-quality massage RCTs with a large sample size and multicentre design are urgently needed. Blinding is not easily implemented in massage interventional RCTs, especially for double-blind. In this study, we identified a total of 16 trials that reported a double-blind method. Except for 10 trials (e.g., 4 Chinese journal papers and 6 international journal papers) which defined the blinded people as subjects and outcome assessors/statisticians, the remaining 6 trials (e.g., 1 Chinese journal paper and 5 international journal papers) actually blinded both subjects and treatment providers. Among these 6 trials, 2 used oil massage (e.g., oil placebo), 1 adopted sham massage device (e.g., the same mechanical massage technique but with different settings of actual massage and sham massage), and 3 achieved sham control through coded different acupoints and locations. The treatment providers in these trials did not know which massage protocol is the placebo control. Finally, nearly half of the included trials did not report any information about ethical approval. Readers thereby cannot fully evaluate such researches as ethical declarations form a vital integral part of clinical trial [24].

The reporting quality of massage RCTs was assessed by three instruments in this review, including the CONSORT checklist, the CONSORT Extension for NPT checklist, and a self-designed massage-specific checklist. Our checklist of message-specific information was designed to identify the critical elements in the rationale for choosing massage as a therapy, the details of massage techniques, and the design of a control for massage interventions. The overall quality of reporting has been unsatisfactory, although a slight improvement has been seen after the CONSORT was updated and issued in 2010.

With regard to the completeness of the CONSORT items, the average reporting rate was 50% (ranging from 0.2 to 99.8%). 18 items were reported poorly (< 50%), of which most (56%, 10/18) related to key methodological domains, such as trial design (item 3a), sample size (items 7a and 7b), sequence generation (items 8a and 8b), allocation concealment mechanism (item 9), implementation (item 10), blinding (item 11b), and additional analyses (item 12b). Previous studies have indicated that the methodology-related items are crucial for the assessment of bias risk and of the reliability of reported effects [25–27]. Other items, including baseline data (item 15), ancillary analyses (item 18), harms (item 19), limitations (item 20), registration (item 23), protocol (item 24), and funding (item 25) in the “Results” and “Other information” sections, were also reported poorly. Inadequate reporting of trial registration, protocol, and ethical approval significantly compromises the value of massage RCTs, inviting skepticism and criticism [28].

At the same time, we identified a considerable number of NPT Extension items that were missing or incompletely reported; the average reporting rate was only 10%, with a range of 0.04% to 57%. Nonpharmacological treatments frequently involve multicomponent interventions delivered by multiple care providers, and each component or provider may influence the success of the overall intervention. Even though the CONSORT NPT Extension was developed in 2008 and updated in 2017 [12], the completeness of reporting remains insufficient, especially in terms of (1) adherence of participants to interventions; (2) changes to the intervention delivered from what was planned; (3) information about care providers; and (4) clustering by care providers or centers. Similar to previous studies, space constraints in journals, such as Chinese journals without an online appendix, is one reason for inadequate reporting of interventions in the RCTs of massage [29]. In this study, we also identified that the reporting score of the CONSORT and NPT items was lower in Chinese articles than that in the international (English) articles. Together, authors, editors (especially for Chinese journals), and reviewers should be more rigorous in their demands that authors adhere to the reporting standards of CONSORT and NPT items in trials of nonpharmacological treatments.

For massage- specific items, the average reporting rate was 45%, with a range of 1.8% to 94.4%. The least well reported information about massage interventions can be summarised in the following aspects: (1) the rationale of why the massage was being used and why the comparison was selected; (2) some details of procedure, particular in individualised massage treatments, media used (if any), responses sought of patients, and management for adverse events; and (3) the background of treatment provider(s). In practice, massage is very much practitioner and experience-dependent, thus, precise and complete details of the manipulation and related factors are essential for the transparency and replication of trials. Some previous analyses suggested that “post-2010 publication years” can be used as an independent predictor of the high reporting quality of RCTs [30], but no great improvement was identified in the included massage RCTs. In this study, we found the reporting score of massage-specific items in Chinese journals was better than that in international (English) journals. We surmise that this is because CM practitioners have a richer experience in conducting complex massage therapies and greater motivation to describe the technique details [31]. Much of traditional CM, since the Shang Dynasty, has been and is based on doctors recording their treatments. Modern doctors have read these earlier accounts, develop different Tuina/Chinese massage schools, know the value of techniques inheritance, and hence are more likely to record those details from their own practice [32].

Given the deficiencies of reporting identified in this study, specific improvements are needed. Previous findings confirm that guidelines do help improve the quality of reporting [17, 33]. Therefore, there are two paths forward: either strengthen reporting of the CONSORT and NPT Extension guidelines, or develop a series of standard reporting items specifically relevant to RCTs with massage interventions as another independent Extension to the general CONSORT 2010 statement. Taking the second route, our working group has initiated and registered the “STandards for Reporting Interventions in Clinical Trials Of Tuina/Massage (STRICTOTM): Extending the CONSORT Statement” on the EQUATOR (Enhancing the QUAlity and Transparency Of health Research) Network [34]. We intend to complete this reporting guideline at the end of 2021.

This study has some limitations. First, this review identified massage RCTs published up to 22 June 2020 in the targeted eight databases. Any records which had not been included in these databases by that cut-off period have not been included. In addition, we included only articles in English and Chinese because of language limitations. As such, we may not have captured otherwise eligible trials published in other languages. Second, we assessed each item with a ‘‘1’’ or ‘‘0’’ score according to whether the author had reported the detailed information listed in the proposed items. All incomplete reporting (e.g., partial and absent) was given as “0”; and some “not applicable” reporting (e.g., open-label without blinding) was categorized as “1”. This over-simplifies the actual situation. Third, we did not assess the methodology quality (e.g., using a Cochrane tool) of each included trial as the primary objective of this study focused on the reporting characteristics. Although the results of this review may not necessarily be comprehensive, we do believe that the general trends indicated by this study are valid.

## Conclusion

Without complete and transparent reporting of how a massage RCT was designed and implemented, it is difficult for readers to assess the reliability and validity of trial findings. Although the CONSORT Statement and NPT Extension are key tools through which adequate reporting can be achieved, the quality of massage RCTs still needs improvement in terms of meeting quality standards and sufficient reporting of massage-specific information. Development of the reporting guideline for massage RCTs, as an extension of CONSORT, should be an effective strategy to improve the current situation.

## Supplementary Information

**Additional file 1.** Search strategy. S2. Quality assessment rules of included trials.

## Data Availability

The data used for this study are included in the manuscript and supplementary file.

## Abbreviations

RCTs: Randomised controlled trials
CONSORT: Consolidated Standards of Reporting Trials
NPT: Nonpharmacologic treatments
MT: Massage therapy
CM: Chinese medicine
CAM: Complementary and alternative medicine
EBM: Evidence-based medicine
AMED: Allied and Complementary Medicine
CNKI: The China National Knowledge Infrastructure
WHO ICTRP: World Health Origination International Clinical Trials Registry Platform
CHM: Chinese herbal medicine
SD: Standard deviations
EQUATOR: Enhancing the QUAlity and Transparency Of health Research
IF: Impact factor
