# Supplementary material for: Assessment of the reporting quality of randomised controlled trials of massage
Source: Chin Med. 2021 Jul 28;16:64. doi: 10.1186/s13020-021-00475-6 (PMC8317306; doi:10.1186/s13020-021-00475-6)
Supplement: Supplementary file 1 — Additional file 1. Search strategy. S2. Quality assessment rules of included trials. [file 13020_2021_475_MOESM1_ESM.pdf]

Additional file 1: S1. Search strategy.

Search Strategy for English databases:

Database: EBM Reviews - Cochrane Database of Systematic Reviews <2005 to June 17, 2020>, EBM Reviews - ACP Journal Club <1991 to May 2020>, EBM Reviews - Database of Abstracts of Reviews of Effects <1st Quarter 2016>, EBM Reviews - Cochrane Clinical Answers <May 2020>, EBM Reviews - Cochrane Central Register of Controlled Trials <May 2020>, EBM Reviews - Cochrane Methodology Register <3rd Quarter 2012>, EBM Reviews - Health Technology Assessment <4th Quarter 2016>, EBM Reviews - NHS Economic Evaluation Database <1st Quarter 2016>, AMED (Allied and Complementary Medicine) <1985 to June 2020>, Embase <1974 to 2020 June 19>, Ovid MEDLINE(R) <1946 to June 19, 2020>
----
1 (((Tui na or tuina) adj3 (Chinese massage or An mo)) or traditional Chinese medicine or massage).mp.
2 (((manual or manipulative or manual therap*) adj3 (soft tissue therap* or soft tissue massage)) or massage).mp.
3 (((acupressure or acupunt) adj3 (meridian or points)) or acupunts massage or points massage or acumassage or pressure massage).mp.
4 ((oil massage or herbal oil massage or sesame oil massage or mustard oil massage or mineral oil massage or sunflower oil massage or coconut oil massage or safflower oil massage or aromatherapy massage) adj3 (oils or lavender essential oil or aroma)).mp.
5 (((infant massage or paediatric massage or baby massage) adj3 (infant or child or baby or paediatrics)) or chiropractics or kneading or pinching massage).mp. [mp=ti, ot, ab, tx, kw, ct, sh, hw, tn, dm, mf, dv, fx, dq, nm, kf, ox, px, rx, an, ui, sy]
6 (((reflexotherapy or reflexion) adj3 (reflexology massage or reflex* therapy)) or therapeutic touch or physical touch or physical contact).mp.
7 (((foot massage or hand massage or head massage or abdominal massage or facial massage or breast massage or back massage) adj3 (skin massage or body massage or muscle massage)) or stroke massage or perineal massage or uterine massage or oral massage or ocular massage).mp.
8 (((Swedish massage or swedish) adj3 (Thai Massage or traditional Thai massage)) or Malay massage or ice massage or hot stone massage or cupping massage or Gua Sha massage or dry massage).mp.
9 (((compression or pressure) adj3 (swing or finger-pushing or kneading or rolling)) or friction or rubbing or pushing or gliding or wiping).mp.
10 (((joint manipulation or rotation or pulling) adj3 (pinching or spine pinching)) or vibration or percussion or grasping).mp.
11 or/1-10
12 randomized controlled trial or controlled clinical trial or randomised controlled trials or random allocation or clinical trials or clinical trial {Including Limited Related Terms}
13 and/11-12

****

Search strategy for CNKI:
SU=('临床试验'+'随机'+'对照'+'随机试验'+'随机对照试验'+'临床研究') and SU=('推拿'+'按摩'+'中医推拿'+'手法'+'按揉'+'捏脊'+'小儿推拿'+'摩腹'+'穴位按摩'+'穴位'+'经络'+'穴位按压'+'点穴'+'经穴推拿'+'指压'+'按压'+'掐压'+'揉压')

Search strategy for VIP database:
(M=临床试验+M=随机+M=对照+M=随机试验+M=随机对照试验+M=临床研究)* (M=推拿+M=按摩+M=中医推拿+M=手法+M=按揉+M=捏脊+M=小儿推拿+M=摩腹+M=穴位按摩+M=穴位+M=经络+M=穴位按压+M=点穴+M=经穴推拿+M=指压+M=按压+M=掐压+M=揉压)

Search strategy for Wanfang database:
(“临床试验”*”随机”*”对照”*”随机试验”*”随机对照试验”*”临床研究”)+(“推拿”*”按摩”*”中医推拿”*”手法”*”按揉”*”捏脊”*”小儿推拿”*”摩腹”*”穴位按摩”*”穴位”*”经络”*”穴位按压”*”点穴”*”经穴推拿”*”指压”*”按压”*”掐压”*”揉压”)

S2. Quality assessment rules of included trials.

Table 1. Assessment rules for the CONSORT and CONSORT for NPT items

Section/topic	Item number and description	Definition of "yes" (scored as "1") or "no" (scored as "0")	Example
Title and abstract	1a. Identification as a randomised trial in the title	It is considered "yes" if the reader can identify the word of "randomised/randomized" in the title.	*Effects of abdominal massage in management of constipation—A randomized controlled trial*
	1b. Structured summary of trial design, methods, results, and conclusions (for specific guidance see CONSORT for abstracts)	It is considered "yes" if the abstract reporting includes objective/background, methods, results, and conclusion/discussion (whether one paragraph, four-structured, or other formats), and the main format is in accordance with the CONSORT for abstracts.	*Question: Does massage relieve pain in the active phase of labour? Design: Randomised trial with concealed allocation, assessor blinding for some outcomes, and intention-to-treat analysis. Participants: 46 women pregnant at > 37 weeks gestation with a single fetus, with spontaneous onset of labour, 4–5 cm of cervical dilation, intact ovular membranes, and no use of medication after admission to hospital. Intervention: Experimental group participants received a 30-min lumbar massage by a physiotherapist during the active phase of labour. A physiotherapist attended control group participants for the same period but only answered questions. Both groups received routine perinatal care. Outcome measures: The primary outcome was pain severity measured on a 100 mm visual analogue scale. Secondary outcomes included the Short Form McGill Pain Questionnaire, pain location, and time to analgesic medication use. After labour, a blinded researcher also recorded duration of labour, route of delivery, neonatal outcomes, and the participant's satisfaction with the physiotherapist during labour. Results: At the end of the intervention, pain severity was 52 mm (SD 20) in the experimental group and 72 mm (SD 15) in control group, which was significantly different with a mean difference of 20 mm (95% CI 10 to 31). The groups did not differ significantly on the other pain-related outcome measures. Obstetric outcomes were also similar between the groups except the duration of labour, which was 6.8 hr (SD 1.6) in the experimental group and 5.7 hr (SD 1.5) in the control*

			group, mean difference 1.1 hr (95% CI 0.2 to 2.0). Patients in both groups were satisfied with the care provided by the physiotherapist. Conclusion: Massage reduced the severity of pain in labour, despite not changing its characteristics and location.
	1b NPT. When applicable, report eligibility criteria for centers where the intervention is performed and for care providers	It is considered "yes" if any criteria-related information of study setting(s) and/or treatment provider(s) was included in the reporting of abstract.	① *Abstract: Design: Parallel-group randomized, controlled trial. Randomization was computer-generated, with centralized allocation concealment…… Setting: An integrated health care delivery system in the Seattle area.*<br>② *Abstract:……The intervention, Qigong Sensory Training (QST), is a qigong massage intervention based in Chinese medicine. It is two-pronged: Trainers work with children directly 20 times over 5 months, and parents give the massage daily to their children. Improvement was evaluated in two settings—preschool and home—by teachers (blind to group) and parents.*<br>③ *Abstract: Experimental group participants received a 30-min lumbar massage by a physiotherapist during the active phase of labour. A physiotherapist attended control group participants for the same period but only answered questions. Both groups received routine perinatal care.*
	1b NPT. Report any important changes to the intervention delivered from what was planned	It is considered "yes" if 1) any changes between protocol and actual implementation were reported; or 2) a statement of adherence to the planned protocol.	①*……The massage treatment was modeled after the Infant Massage USA (Springfield, Virginia) protocol and modified for preterm infants by eliminating massage of the abdomen……*<br>② *Swedish massage was provided in a standardized fashion and included effleurage and pe´trissage strokes……*
Introduction			
Background and objectives	2a. Scientific background and explanation of rationale	It is considered "yes" if the paper includes the "Introduction/Background" section and descript the research background and rationale of	*Introduction: Massage is one of the most popular complementary and alternative medical therapies for neck and back pain (1), conditions that account for more than one third (2) of the more than 100 million annual visits to massage therapists in the United States (3). Almost all massage therapists in the United States use Swedish massage techniques aimed at relaxation, but only a minority take courses in such techniques as structural massage for treatment of*

		studied diseases and interventions.	*chronic low back pain. Recent reviews have found limited evidence that massage is an effective treatment of chronic back pain (4, 5), and no studies have compared relaxation massage with structural massage, which focuses on correcting soft-tissue abnormalities. We therefore conducted a trial to determine whether relaxation massage reduces pain and improves function in patients with chronic low back pain and compared relaxation and structural massage for treating this condition.*
	2b. Specific objectives or hypotheses	It is considered "yes" if the study objective was provided in the "Introduction" section of the paper.	*Introduction: This study investigates the effects of abdominal massage on gastrointestinal functions and laxative intake on persons with constipation. The hypothesis was that abdominal massage could affect the severity of gastrointestinal symptoms, number of bowel movements, time to defecate, faeces consistency, quantity of faeces, and decrease in laxative use without increased fluid and fibre intake or increased physical activity.*
Methods			
Trial design	3a. Description of trial design (such as parallel, factorial) including allocation ratio	It is considered "yes" if 1) the trial design was specified in the "Methods" and 2) allocation ratio was also provided. If the partial reporting without allocation ratio, this item should be scored as "0".	*Methods: This prospective, non-blinded, and randomized controlled trial (1:1) was conducted in Sweden between January 2005 and March 2007……*
	3a NPT. When applicable, how care providers were allocated to each trial group	It is considered "yes" if any information about treatment providers in each group was reported in the "study design".	*Both massage treatments and sham interventions were conducted by licensed therapists, and they were trained to provide the same answers to the patient' questions. In addition, they were advised to be quiet during the treatment.*
	3b. Important changes to methods after trial commencement (such as eligibility criteria), with	It is considered "yes" if 1) any changes included in the trial; or 2) statement of there is no changes in the methods.	*① Although there is no evidence that massage of the abdomen is associated with the development of necrotizing enterocolitis or other abdominal injury, we eliminated massage of the abdomen as a precautionary measure. The massage protocol consisted of the application of 6 soft-tissue compression strokes to the following areas of the supine infant:……*

	reasons		*② The criteria of patients recruitment is based on the protocol (Appendix 2)…….*
Participants	4a. Eligibility criteria for participants	It is considered "yes" if inclusion and exclusion criteria for participants were reported in the article. If some paper provided this content in an available protocol, it also be scored as "1". If some trials did not provide the exclusion criteria, it should be scored as "0".	*Inclusion and exclusion criteria of patients with back pain in past five years*<br>*Inclusion criteria: to identify those with significant recurrent pain or chronic pain*<br>*-Presentation in primary care with low back pain more than three months previously (to exclude first episodes)*<br>*-Currently scoring 4 or more on the Roland disability scale*<br>*-Current pain for three or more weeks (to exclude recurrence of short duration)*<br>*Exclusion criteria*<br>*-Previous experience of Alexander technique*<br>*-Patients under 18 and over 65 (serious spinal disease more likely)*<br>*-Clinical indicators of serious spinal disease*<br>*-Current nerve root pain (below knee in dermatomal distribution), previous spinal surgery, pending litigation (outcome may be different, groups too small to analyse)*<br>*-History of psychosis or major alcohol misuse (difficulty completing outcomes)*<br>*-Perceived inability to walk 100 m (exercise difficult).*
	4a NPT. When applicable, eligibility criteria for centers and for care providers	It is considered "yes" if any information about the criteria of centres or care providers was provided. The descriptions can be separated in different paragraph but all in the "Methods".	*Participants were recruited from the women admitted to the Reference Center of Women's Health of Ribeirão Preto-MATER, state of São Paulo, Brazil, between September 2009 and May 2010. This is a 40-bed unit that serves a mean of 3600 patients per year in Brazil's public health system……. The two therapists involved in the intervention and data collection had both specialised in women's health since early 2008……*
	4b. Settings and locations where the data were collected	It is considered "yes" if the names and locations (at least report the country/region) of centres were provided in the Methods.	*We recruited 64 general practices in the south and west of England in two centres (Southampton and Bristol) on the basis of geographical availability of teachers of the Alexander technique and massage therapists…….*
Interventions	5. Interventions for each	-	-

	group with sufficient details to allow replication, including how and when they were actually administered		
	5a NPT. Precise details of both the experimental treatment and comparator	-	-
	5b NPT. Description of the different components of the interventions and, when applicable, description of the procedure for tailoring the interventions to individual participants.	-	-
	5c NPT. Details of whether and how the interventions were standardised.	It is considered "yes" if the article reported that 1) the interventions were standardised; or 2) some changes included with reasons; or 3) specific details were provided in an available protocol.	*Eight licensed massage therapists with at least 5 years of experience were trained in the study protocol and provided massage treatments in the research clinic at Group Health…….*
	5d NPT. Details of whether and how adherence of care	It is considered "yes" if the article reported that 1) training arrangement for care providers; 2)	*Massage was provided by 27 licensed therapists with at least 5 years of experience who were comfortable following the study protocol and had experience in the permitted techniques. Therapists received 1.5 days of protocol training. Treatment fidelity was promoted by monitoring*

	providers to the protocol was assessed or enhanced	how to assess the adherence of care providers to the protocol.	*treatment forms that the therapists completed at each visit, along with corrective feedback and a midstudy meeting.*
	5e NPT. Details of whether and how adherence of participants to interventions was assessed or enhanced	It is considered "yes" if the any information about patient's adherence assessment was provided.	*We defined "adherence" as completion of at least 8 visits. At each visit, therapists could recommend up to 3 home exercises from a predefined list of 7 exercises, 6 of which were common to both treatments…….*
Outcomes	6a. Completely defined pre-specified primary and secondary outcome measures, including how and when they were assessed	It is considered "yes" if both name and evaluation methods of outcomes were reported. If some partial reporting in the all outcomes (primary and secondary), it should be scored as "0".	*The first primary outcome measure was disability, measured using the Roland Morris disability questionnaire……. Secondary outcome measures were quality of life, measured using the short form 36,……*
	6b. Any changes to trial outcomes after the trial commenced, with reasons	It is considered "yes" if 1) both changes and reasons were reported; or 2) reported as "no change". If there is no clear reporting and reader cannot identify whether there include changes, it should be scored as "0".	*The analysis plan was agreed in advance by the trial management group. The primary analysis was ……. for the primary outcome between groups (Roland disability score) and for the secondary outcomes…….*
Sample size	7a. How sample size was determined	It is considered "yes" if specific procedure of sample size	*A sample size calculation was performed with regard to the gastrointestinal-specific research tool GSRS (Svedlund et al., 1988). Mean item score for a normal population was 1.53 (Dimena ̈s*

		calculation was provided. If only a "number" of sample size but without any rationale description, it should be scored as "0".	*et al., 1996) and in a population with constipation the mean item score was 2.63 (Glia and Lindberg, 1997). The mean difference between a normal population and a population with constipation is then 1.1. In order to detect even small differences the power calculation is based on a GSRS mean difference of 0.6. A sample size of 30 in each group will have 80% power to detect a difference in GSRS mean score of 0.6 with standard deviation of 0.8 and with a significance level of 5%.*
	7a NPT. When applicable, details of whether and how the clustering by care providers or centers was addressed	It is considered "yes" if the article reported that 1) any pre-calculation of sample size in each centre; or 2) any information about clustering by centres and care providers.	*① Sample size: ……The total number is 256. We anticipated that each of the participating centres would treat approximately 1.5 eligible patients per month…….*<br>*② We assessed the statistical significance of clustering by therapist, teacher, and practice, and if these were not significant we did not allow for clustering in the models.*
	7b. When applicable, explanation of any interim analyses and stopping guidelines	It is considered "yes" if the article mentioned 1) interim analyses (whether have or no); or 2) stopping guidelines; or 3) explanation of stopping guidelines relative with interim analyses.	①*An independent and blinded data monitoring and safety committee assessed the interim analyses……*<br>②*Termination criteria for the trial: Presence of serious adverse effect or completion of all follow-up visits.*
Sequence generation	8a. Method used to generate the random allocation sequence	It is considered "yes" if the generate method was provided. It should be noted that if the generate method of random was not actual randomised (very common in Chinese papers), such as coin toss, odd and even	*A statistician had prepared a secure program using computer generated random numbers so that the next allocation could not be guessed……*

		numbers, patients sequence, etc. these should be scored as “0”.	
	8b. Type of randomisation; details of any restriction (such as blocking and block size)	It is considered “yes” if the articles reported the details of randomisation.	*For each practice contributing 10 patients a block of eight number existed, and two were added from a block that supplied four other practices. Practices were not told how many patients would be recruited to each trial group or informed of the block randomisation. When possible each practice was matched to two Alexander technique teachers.*
Allocation concealment mechanism	9. Mechanism used to implement the random allocation sequence (such as sequentially numbered containers), describing any steps taken to conceal the sequence until interventions were assigned	It is considered “yes” if the allocation concealment methods were reported.	*The participants received a numbered and sealed envelope and opened the envelope in the presence of the administrative assistant…….*
Implementation	10. Who generated the random allocation sequence, who enrolled participants, and who assigned participants to interventions	It is considered “yes” if different investigators were defined, including generate the random number, enroll subjects, and assign the interventions.	*At the baseline appointment, after informed written consent had been obtained, participants were randomised to one of eight groups by the practice nurse telephoning the central coordinating centre in Southampton (table 1 and appendix on bmj.com). A statistician had prepared a secure program using computer generated random numbers……Massage therapists provided the treatments according to the protocol……*
Blinding	11a. If done, who was blinded after assignment to interventions (for example, participants,	It is considered “yes” if the article reported that 1) blinding type (single or double) and blinded people (e.g., patients, treatment	*Therapists were not blinded to the type of massage that they provided. Participants knew whether they received massage but were blinded to type; usual care recipients were aware that they had enrolled in a trial of massage. Study personnel assessing trial outcomes were blinded to study assignment.*

	care providers, those assessing outcomes) and how	providers, etc.); or 2) open-label design (not applicable for this item). If the blinding was used in the trial, partial reporting of type and who was blinded should be scored as "0". If the readers cannot identify whether blinding was adopted in the trial, this should be scored as "0".	
	11a NPT. If done, who was blinded after assignment to interventions (e.g., participants, care providers, those administering co-interventions, those assessing outcomes) and how	It is considered "yes" if both blinding type (single or double) and blinded people (e.g., patients, treatment providers, etc.) were reported in the article. The situations of partial reporting and not applicable (open-label) were scored as "0".	*This is a perspective, randomised, double blinded clinical study……, Both patients and therapists are blinded…….The massage and control treatments were performed behind a privacy screen by a licensed massage therapist……. Both massage and control procedures were administered behind a privacy screen to maintain "masking" of the infant's study assignment to parents and NICU clinical staff.*
	11b. If relevant, description of the similarity of interventions	It is considered "yes" if the article provided a description of similarity between intervention and placebo. This is only assessed in the trials with blinding design. The open-label trials should be scored as "0".	*Sham massage was administered by an instructed colleague according to a standardised protocol in the same treatment room under the same conditions. In this procedure, both hands were placed with moderate pressure for about 3 min on 10 different body areas. The duration of the sham massage and the additional rest period was identical in all groups. The therapists had undergone training in rhythmic massage and were certified by the German Professional Association……*

	11c NPT. If blinding was not possible, description of any attempts to limit bias	It is considered "yes" if the article reported any information about bias control and assessment for the open-label design.	*To reduce the bias between patients with different type of massage in two groups, the nurses (treatment provider) should not provide any treatment-related information to the subjects. If some questions proposed by the patients, nurses are trained to use a standardised protocol ……*
Statistical methods	12a. Statistical methods used to compare groups for primary and secondary outcomes	It is considered "yes" if statistical methods for both primary and secondary outcomes were provided.	*Analyses were conducted according to the original randomized treatment assignment regardless of adherence to protocol. Analyses were conducted by using regression through generalized estimating equations with an independent working correlation structure and robust SE estimates taking into account multiple outcomes per participant. Follow-up times were treated as categorical variables using dummy variables for each treatment, each time point, and all 2-way interactions between follow-up time and treatment. Adjusted models included baseline covariates that were prespecified, were imbalanced at baseline (that is, potential confounders), or were associated with a primary outcome (that is, precision variables): age, group, sex, baseline RDQ and symptom bother someness scores, education level, body mass index, type of work, original cause of back pain, more than 7 days of reduced activities because of back pain, and medication use in the previous week…….*
	12a NPT. When applicable, details of whether and how the clustering by care providers or centers was addressed	It is considered "yes" if any statistical methods was pre-defined to consider "clustering by centre, or care providers".	*For categorical demographic and disease characteristics, the Mantel-Haenszel test with stratification by centre was used. For continuous variables, an analysis of variance (ANOVA) model with effects for treatment, centre and treatment-by-centre interaction was used. If the treatment-by-centre interaction term was not significant, it was dropped from the model…….*
	12b. Methods for additional analyses, such as subgroup analyses and adjusted analyses	It is considered "yes" if additional statistical analysis was reported, such as subgroup analysis, regression analysis, sensitivity analysis, etc.	*We prespecified the adjusted analysis as the primary analysis. For continuous and binary outcome measures, we applied linear and modified Poisson regression, respectively, with robust SEs. Modified Poisson regression allows estimation of relative risks for nonrare outcomes using Poisson regression and corrects the misspecification of the variance using robust SEs in a generalized estimating equation framework………*

Results			
Participant flow (a diagram is strongly recommended)	13a. For each group, the numbers of participants who were randomly assigned, received intended treatment, and were analysed for the primary outcome	It is considered “yes” if 1) study flow chart was provided; or 2) detailed description was provided. Partial reporting should be scored as “0”.	*Figure 1.* **Study flow diagram.**<br>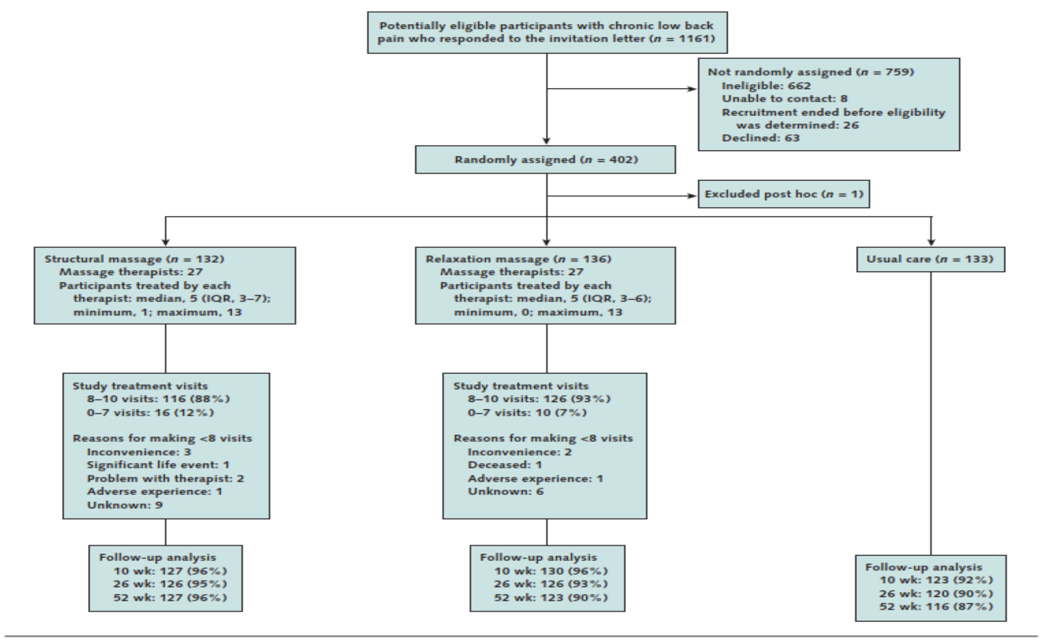

	13a NPT. The number of care providers or centers performing the intervention in each group and the number of patients treated by each care provider or in each center	It is considered "yes" if 1) centre-based results were provided; or 2) specific number in each group by different care providers was metioned.	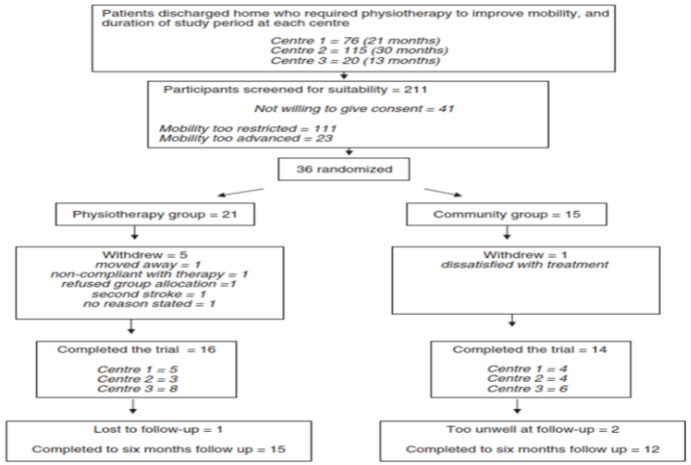<br>**Figure 1** Flow of participants through the trial.
	13b. For each group, losses and exclusions after randomisation, together with reasons	It is considered "yes" if losses and exclusions with reasons in each group were reported.	*Two participants withdrew before baseline because of lack of time; they were excluded. Three participants in each group withdrew after baseline; one in the intervention group because of troublesome blood pressure dip with numbness in hands and feet after the first session and two in the control group where one died and one reported lack of time. Three participants withdrew after week 4; two in the intervention group (one developed diverticulitis and one went on vacation) withdrew. One participant in the control group went on vacation…….*
	13c NPT. For each group, the delay between randomisation and the initiation of the intervention	It is considered "yes" if the article mentioned if there any delay between *randomisation and the initiation of the intervention*	*…… After the randomisation allocation, the patient was given a coded treatment protocol immediately……*
Recruitment	14a. Dates defining the periods of recruitment and follow-up	It is considered "yes" if the article reported 1) recruitment period and specific dates; and 2) the period of follow-up (if any).	*We recruited patients from 8 July 2002 to 22 July 2004……Follow-up of study outcomes was conducted through mailed questionnaire at 12 and 24 weeks and through phone interview by a blinded research assistant at 4, 8, 16, and 20 weeks…….*

<table>
<tr><td></td><td></td><td>Some papers that did not report the specific dates should be scored as “0”.</td><td></td></tr>
<tr><td></td><td>14b. Why the trial ended or was stopped</td><td>It is considered “yes” if the article reported 1) the whole trial was ended with reasons; or 2) any centre(s) was stopped with reasons; or 3) the trial was completion.</td><td>We enrolled 1717 participants in 11 centres (figure 1). We intended to enrol 150 women at each of 14 sites, but three of the selected sites were unable to join the trial. Among the participating centres, some (Melbourne and San Francisco) had slower enrolment than expected……</td></tr>
<tr><td>Baseline data</td><td>15. A table showing baseline demographic and clinical characteristics for each group</td><td>It is considered “yes” if the article reported the baseline results (with Table).</td><td>There were 50 women and eight men who participated (mean age of 63.7 year). No significant differences were found at baseline between the intervention and control groups regarding sex, age, marital status, housing, physical ability and laxative use (Table 1).
Table 1
Baseline characteristics of participants.
<table>
<tr><th>Baseline characteristics</th><th>Intervention, n = 29</th><th>Control, n = 29</th><th>p-Values</th></tr>
<tr><td>Female</td><td>26 (90)</td><td>24 (83)</td><td></td></tr>
<tr><td>• Male</td><td>3 (10)</td><td>5 (17)</td><td>.71</td></tr>
<tr><td>• Age (year)</td><td>64 ± 10.4 (47–85)</td><td>63 ± 10.6 (36–79)</td><td>.60</td></tr>
<tr><td>Marital status</td><td></td><td></td><td>1.0</td></tr>
<tr><td>• Living alone</td><td>10 (36)</td><td>10 (35)</td><td></td></tr>
<tr><td>• Married/cohabitant</td><td>18 (64)</td><td>19 (65)</td><td></td></tr>
<tr><td>Housing</td><td></td><td></td><td>1.0</td></tr>
<tr><td>• Living at home</td><td>27 (93)</td><td>26 (90)</td><td></td></tr>
<tr><td>• Nursing home</td><td>2 (7)</td><td>3 (10)</td><td></td></tr>
<tr><td>Occupation</td><td></td><td></td><td>1.0</td></tr>
<tr><td>• Pensioner</td><td>15 (48)</td><td>17 (55)</td><td></td></tr>
<tr><td>• Working</td><td>11 (38)</td><td>8 (28)</td><td></td></tr>
<tr><td>• Sick leave</td><td>4 (14)</td><td>4 (14)</td><td></td></tr>
<tr><td>• Unemployed</td><td>0</td><td>1 (3)</td><td></td></tr>
<tr><td>Physical ability</td><td></td><td></td><td>1.0</td></tr>
<tr><td>• Walking</td><td>26 (90)</td><td>25 (86)</td><td></td></tr>
<tr><td>• Wheelchair</td><td>3 (10)</td><td>4 (14)</td><td></td></tr>
<tr><td>Laxantia use</td><td></td><td></td><td>.79</td></tr>
<tr><td>• Occasionally</td><td>13 (45)</td><td>11 (38)</td><td></td></tr>
<tr><td>• Daily</td><td>16 (55)</td><td>18 (62)</td><td></td></tr>
</table></td></tr>
</table>

<table>
<tr>
<td></td>
<td>15 NPT. When applicable, a description of care providers (case volume, qualification, expertise, etc.) and centers (volume) in each group</td>
<td>It is considered “yes” if the centre-based (or clustering by providers) data regarding baseline results was provided.</td>
<td>
<table>
<tr><th></th><th>Telemedicine (n=465)</th><th>Standard care (n=444)</th></tr>
<tr><td>Centre</td><td></td><td></td></tr>
<tr><td>Maastricht University Medical Centre</td><td>133 (29%)</td><td>131 (30%)</td></tr>
<tr><td>Leiden University Medical Centre</td><td>144 (31%)</td><td>152 (34%)</td></tr>
<tr><td>Zuyderland Medical Centre, Sittard</td><td>117 (25%)</td><td>102 (23%)</td></tr>
<tr><td>St Antonius Hospital, Nieuwegein</td><td>71 (15%)</td><td>59 (13%)</td></tr>
<tr><td>Sex</td><td></td><td></td></tr>
<tr><td>Men</td><td>194 (42%)</td><td>180 (41%)</td></tr>
<tr><td>Women</td><td>271 (58%)</td><td>264 (59%)</td></tr>
<tr><td>Age at diagnosis (years)</td><td>30·7 (13·5)</td><td>30·4 (13·6)</td></tr>
<tr><td>Age at inclusion (years)</td><td>44·0 (14·1)</td><td>44·1 (14·2)</td></tr>
<tr><td>Disease duration (years)</td><td>12·8 (10·4)</td><td>13·1 (10·8)</td></tr>
<tr><td>Phenotype</td><td></td><td></td></tr>
<tr><td>Crohn’s disease*</td><td>282 (61%)</td><td>262 (59%)</td></tr>
<tr><td>Ileal</td><td>87 (31%)</td><td>68 (26%)</td></tr>
<tr><td>Colonic</td><td>67 (24%)</td><td>63 (24%)</td></tr>
<tr><td>Ileocolonic</td><td>128 (45%)</td><td>131 (50%)</td></tr>
<tr><td>Upper gastrointestinal modifier</td><td>34 (12%)</td><td>26 (10%)</td></tr>
<tr><td>Non-penetrating, non-stricturing, B1</td><td>169 (60%)</td><td>152 (58%)</td></tr>
<tr><td>Stricturing, B2</td><td>76 (27%)</td><td>70 (27%)</td></tr>
<tr><td>Penetrating, B3</td><td>37 (13%)</td><td>40 (15%)</td></tr>
</table>
Data are n (%) or mean (SD). *The number of patients with Crohn’s disease is used as the denominator for the percentages of each subtype of Crohn’s disease. †The number of patients with ulcerative colitis is used as the denominator for the percentages of each subtype of ulcerative colitis.
Table 1: Baseline characteristics
</td>
</tr>
<tr>
<td>Numbers analysed</td>
<td>16. For each group, number of participants (denominator) included in each analysis and whether the analysis was by original assigned groups</td>
<td>It is considered “yes” if the article reported the analysed number in each group.</td>
<td>The analysed number of patients in the two groups are provided in Fig 2…….</td>
</tr>
<tr>
<td>Outcomes and estimation</td>
<td>17a. For each primary and secondary outcome, results for each group, and the estimated effect</td>
<td>It is considered “yes” if the results data were standardised reported, whether in mean (SD) or 95% CI, both formats were correct based</td>
<td></td>
</tr>
</table>

<table>
<tr>
<td></td>
<td>size and its precision (such as 95% confidence interval)</td>
<td rowspan="2">on the types of data.</td>
<td rowspan="2">
Table 3 | Outcomes at three months after randomisation. Values are mean differences compared with control group (95% confidence intervals) and P values, unless stated otherwise
<table>
<tr><th rowspan="2">Outcomes</th><th rowspan="2">Mean (SD) control (Alexander technique factor)*</th><th colspan="3">Mean difference compared with control, P value</th><th rowspan="2">Mean (SD) control (exercise factor*)</th><th rowspan="2">Mean difference compared with control: exercise</th></tr>
<tr><th>Massage</th><th>6 lessons in Alexander technique</th><th>24 lessons in Alexander technique</th></tr>
<tr><td colspan="7">Primary outcomes</td></tr>
<tr><td>Roland disability score† (n=469)</td><td>9.34 (4.76)</td><td>−1.96 (−0.74 to 3.18), P=0.002</td><td>−1.71 (−2.95 to −0.47), P=0.007</td><td>−2.91 (−4.16 to 1.66), P<0.001</td><td>8.35 (4.75)</td><td>−0.90 (−1.76 to 0.04), P=0.04</td></tr>
<tr><td>Median (95% CI) No of days with back pain in past 4 weeks (n=405)‡</td><td>24 (21 to 27)</td><td>−13 (−18 to −8), P<0.001</td><td>−11 (−16 to −6), P<0.001</td><td>−16 (−21 to −11) P<0.001</td><td>17 (15 to 19)</td><td>−6 (−9 to −3), P<0.001</td></tr>
<tr><td colspan="7">Secondary outcomes</td></tr>
<tr><td>SF-36: quality of life physical§ (n=403)</td><td>54.9 (16.5)</td><td>2.57 (−2.20 to 7.34), P=0.290</td><td>4.39 (−0.40 to 9.19), P=0.072</td><td>7.5 (2.60 to 12.4), P=0.003</td><td>56.6 (16.5)</td><td>3.0 (−0.22 to 6.23), P=0.068</td></tr>
<tr><td>SF-36: quality of life mental§ (n=398)</td><td>62.5 (17.3)</td><td>−0.37 (−5.37 to 4.64), P=0.886</td><td>2.88 (−2.18 to 7.94), P=0.264</td><td>3.36 (−1.82 to 8.53), P=0.203</td><td>62.5 (17.2)</td><td>4.04 (0.65 to 7.43), P=0.020</td></tr>
<tr><td>Modified enablement scale¶ (n=386)</td><td>3.78 (1.15)</td><td>1.43 (1.10 to 1.76), P<0.001</td><td>1.45 (1.11 to 1.80), P<0.001</td><td>1.82 (1.47 to 2.16), P<0.001</td><td>4.80 (1.15)</td><td>0.41 (0.17 to 0.64), P=0.001</td></tr>
<tr><td>Von Korff overall** (n=412):</td><td>3.89 (1.71)</td><td>−0.13 (−0.60 to 0.35), P=0.597</td><td>−0.18 (−0.66 to 0.30), P=0.462</td><td>−0.47 (−0.96 to 0.02), P=0.061</td><td>3.83 (1.70)</td><td>−0.26 (−0.59 to 0.07), P=0.126</td></tr>
<tr><td>Von Korff disability††</td><td>3.27 (1.90)</td><td>0.00 (−0.51 to 0.52), P=0.993</td><td>0.00 (−0.52 to 0.52), P=0.990</td><td>−0.22 (−0.74 to 0.31), P=0.170</td><td>3.33 (1.90)</td><td>−0.25 (−0.61 to 0.11), P=0.170</td></tr>
<tr><td>Von Korff pain††</td><td>4.62 (1.85)</td><td>−0.41 (−0.91 to 0.09), P=0.110</td><td>−0.48 (−0.98 to 0.028), P=0.064</td><td>−0.75 (−1.26 to −0.24), P=0.004</td><td>4.39 (1.84)</td><td>−0.32 (−0.66 to 0.03), P=0.074</td></tr>
<tr><td>Deyo troublesomeness‡‡ (n=449)</td><td>3.09 (0.72)</td><td>−0.22 (−0.41 to −0.03), P=0.026</td><td>−0.20 (−0.40 to 0.01), P=0.039</td><td>−0.33 (−0.52 to −0.13), P=0.001</td><td>2.98 (0.72)</td><td>−0.11 (−0.24 to 0.02), P=0.103</td></tr>
<tr><td>Health transition§§ (n=433)</td><td>3.84(0.91)</td><td>−0.94 (−1.19 to −0.70), P<0.001</td><td>−0.81 (−1.06 to −0.56), P<0.001</td><td>−1.10 (−1.36 to −0.85), P<0.001</td><td>3.23 (0.91)</td><td>−0.22 (−0.39 to −0.05), P=0.013</td></tr>
<tr><td>Fear avoidance for physical activity (n=404)¶¶</td><td>14.2 (5.0)</td><td>−0.58 (−2.0 to 0.86), P=0.432</td><td>−0.80 (−2.25 to 0.64), P=0.276</td><td>−1.93 (−3.41 to −0.45), P=0.011</td><td>14.3 (5.0)</td><td>−2.70 (−3.68 to −1.72), P<0.001</td></tr>
<tr><td>Back health (n=407)***</td><td>3.35 (1.40)</td><td>1.56 (1.16 to 1.96), P<0.001</td><td>1.48 (1.08 to 1.89), P<0.001</td><td>1.84 (1.43 to 2.25), P<0.001</td><td>4.33 (1.40)</td><td>0.53 (0.26 to 0.80), P<0.001</td></tr>
</table>
[illegible]
</td>
</tr>
<tr>
<td></td>
<td>17b. For binary outcomes, presentation of both absolute and relative effect sizes is recommended</td>
</tr>
<tr>
<td>Ancillary analyses</td>
<td>18. Results of any other analyses performed, including subgroup analyses and adjusted analyses, distinguishing pre-specified from exploratory</td>
<td>It is considered "yes" if the article provided 1) additional analysis results (in accordance with the pre-designed statistical methods); or 2) additional analysis results with reasons o clarifications (for those have no pre-designed statistical methods); or 3) no results for the additional analysis because there is no necessary to conduct.</td>
<td>① No significant cluster effects (practice, therapist or teacher) were found, except for enablement, where a practice clustering effect was found, so only these results are presented allowing for clustering.
② Multivariable regression analyses showed only very small effects of differences in baseline values of the HDI, cervical range of motion and algometry on outcome. Therefore, we presented crude estimates in Tables 2 and 3. A subgroup analysis for CTTH participants with co-morbid migraine showed similar results for the primary outcome measures……</td>
</tr>
<tr>
<td>Harms</td>
<td>19. All important harms or unintended effects in each group (for specific</td>
<td>It is considered "yes" if the article reported "adverse effect" or "safety assessment", whether it</td>
<td>Five of 134 (4%) relaxation massage recipients and 9 of 131 (7%) structural massage recipients reported adverse events possibly related to massage, mostly increased pain. One event in the structural massage group (nausea, shortness of breath, and chest pain) was classified as serious</td>
</tr>
</table>

	guidance see CONSORT for harms)	was identified or not. If no consideration of the safety issue canbe found through the article, it should be scored as "0".	*and considered unrelated to treatment.*
Discussion			
Limitations	20. Trial limitations, addressing sources of potential bias, imprecision, and, if relevant, multiplicity of analyses	It is considered "yes" if the article provided a limitation discussion.	*There were several potential limitations to the study. Data collection was limited to 44 preterm infants born at 29-32 weeks' gestation. Our small sample size was offset by: (1) restricting eligibility to medically stable, preterm infants to ensure a more homogeneous cohort in regard to growth and body composition; and (2) using a prospective, longitudinal study design to increase statistical power. Our study was designed to ensure protocol compliance and limit study- related bias by masking to infant treatment assignment of all personnel involved in measurements and testing.*
	20 NPT. In addition, take into account the choice of the comparator, lack of or partial blinding, and unequal expertise of care providers or centers in each group	It is considered "yes" if the article discussed the limitations from these factors proposed in this item. If there is no limitation discussed, it should be scored as "0".	*Participants assigned to usual care were told that they were enrolling in a trial of massage therapy, and they often received no additional treatment. This potential failure of blinding to treatment assignment may have led to less favorable self-assessments of function and symptoms, making massage therapy seem more superior than it really is……*
Generalisability	21. Generalisability (external validity, applicability) of the trial findings	It is considered "yes" if the generalisability of the results were reported in the "Discussion".	*Abdominal massage can complement laxative use for people with constipation when laxatives do not have the desired effect. The massage, however, has a delayed effect that may occur first after a number of weeks and is considered to be a long-term treatment. A prerequisite for the massage treatment is that the person feels comfortable with receiving abdominal massage. This requires a sensitive therapist that can develop a trusting relationship.*
	21 NPT. Generalizability (external validity) of the	It is considered "yes" if the generalisability of the results	*·····the results were obtained in highly specialized centres and all procedures were performed by experienced surgeons, which may limit the generalizability of the results…….*

	trial findings according to the intervention, comparators, patients, and care providers and centers involved in the trial	were discussed from these factors (only one is ok) proposed in this item.	
Interpretation	22. Interpretation consistent with results, balancing benefits and harms, and considering other relevant evidence	It is considered "yes" if the discussions included interpretations of benefits and harms of studied interventions and provided relevant references.	*The mechanisms explaining the beneficial effects of relaxation and structural massage remain unclear. These distinct forms of massage may trigger similar physiologic effects (for example, through local stimulation of tissue or a generalized central nervous system response) or may work through different mechanisms (for example, structural massage may foster beneficial changes in the treated soft tissues, whereas relaxation massage may operate through the central nervous system)…….*
Other information			
Registration	23. Registration number and name of trial registry	It is considered "yes" if both registration number and registry name were reported.	*Trial registration National Research Register N0028108728.*
Protocol	24. Where the full trial protocol can be accessed, if available	It is considered "yes" if the study protocol was reported by an accessible way, such as hyperlink, references, registration platform, or others.	*The study protocol in its final form has been published on the UK Stroke Research Network Web site, where it is available to the public: URL, http://public.ukcrn.org.uk/search/StudyDetail.aspx?StudyID=3279; study ID, 03884521.*
Funding	25. Sources of funding and other support (such as supply of drugs), role of funders	It is considered "yes" if 1) provide d the funding information; or 2) reported no funding(s).	*This trial was funded by the National Center for Complementary and Alternative Medicine (NCCAM) and was approved their Office of Clinical and Regulatory Affairs. The NCCAM did not participate in the research.*

Table 2. Assessment rules for the self-designed massage-specific checklist

Item	Specifics	Definition of "yes"<br>(e.g. scored as "1")	Example
Massage rationale	Q1. Whether the type of massage was reported?	It is considered "yes" if the specific type/style (e.g., adult tuina, infantile tuina, TCM massage, Swedish/Thai/Ice/Aroma massage) of massage was reported. If only reported as a generalized name (massage), it should be scored as "0".	① *We aimed to investigate the effects of infant massage on neonates with jaundice who are also receiving phototherapy.*<br>② *The purpose of this study was to assess the efficacy of Chinese massage (Tui Na) in improving knee extensor and flexor muscle strength in patients with knee OA.*
	Q2. Whether the rationale for selected massage was provided?	It is considered "yes" if the selected rationale/reasons were provided. Partial reporting (only focused on the diseases and interventions but without reporting of selection) should be scored as "0".	① *Anma massage therapy (Japanese massage therapy, AMT) is a popular form of complementary and alternative medicine in Japan. Based on anecdotal information, it has long been used to relieve physical and psychological complaints in healthy persons as well as in persons with cancer-related symptoms……*
	Q3. Whether any information about individualised massage treatment was reported?	It is considered "yes" if 1) the individualised massage treatment was provided with detailed criteria; or 2) reported standardised massage implementation. If the article did not provide any information about the individuliased or standardised, it should be scored as "0".	① *Tuina doctors may adjust the force (light, medium, or heavy) and duration of manipulation according to patient's conditions……*<br>② *For both groups, a treatment protocol was developed to allow sufficient standardization to assist replication, yet was flexible enough to allow individualized treatments based on a Traditional Chinese Medicine (TCM) theory…….*
Details of massage	Q4. Whether the patient posture and/or environment during treatment was mentioned?	It is considered "yes" if the articles reported that 1) treatment environment; or 2) patient posture during treatment. Partial reporting should be scored as "0".	① *The same researcher provided massage therapy to all neonates in the study, and the room temperature was maintained at between 26 °C and 28 °C.*<br>② *Participants wore comfortable clothing in a supine position in*

			bed or in a reclined wheelchair. The supine position was selected over the more traditional prone position since the prone position may be contraindicated for those with spinal cord injury (SCI) and evidence suggested a supine position results in similar responses as a prone position
	Q5. Whether the media (e.g., dosage and manufacturers) used for massage was reported?	It is considered "yes" if 1) media, such as oil, herbs, etc. were used, details should be reported; or 2) massage without any media. For the oil massage, trials with insufficient information about manufacture were scored as "0".	*Patients in the olive oil group received olive oil massage……We used refined, pure, odorless olive oil (Loyeh Ind., Gilan, Iran; health production license No., 47/10794; production serial No., PZS1626815).*
	Q6. Whether the massage points and/or locations were provided?	It is considered "yes" if the specific locations for massage were clearly reported. For acupoints, if no standard or detailed description was provided, it should be scored as "0".	*Each session consisted of pressing and thumb-kneading on eight acupoints that are common local points used to treat knee problems and reduce knee pain; points used were: YinLingquan (SP 9), Xuehai (SP 10), Liang Qiu (ST 34), Heding (EX 31), inside and outside Xiyan (EX 32), Zusanli (ST 36), Yanglingquan (GB 34) and Weizhong (BL 40). Acupoints were located according to the World Health Organization standard acupuncture point location.*
	Q7. Whether the duration, frequency and/or force of massage in per point and/or location was reported?	It is considered "yes" if the treatment sessions with duration, frequency, and/or force for each location were provided. If only the force information was absent, it could be scored as "1" when good reporting in other details.	*① Doctors applied light, medium, or heavy force to Chengjin (BL 56) at participants' musculus gastrocnemius for 2, 5, or 10 min (The force-time combinations for the nine groups are shown in Figure 1).*<br>*② First, the upper segment of sternocleidomastoid muscle was massaged for 5 minutes using PMTMOF, then the middle segment was massaged for 10 minutes, and subsequently the inferior segment for 5 minutes. The entire manipulation lasted for 20*

			minutes with the patient in a recumbent position (atrophy type for 15 minutes). The pendular movement frequency was 220–250 times/min
	Q8. Whether the details of procedure and technique of massage was described?	It is considered "yes" if the article reported the procedure and operation details for Tuina/massage, including manipulation and force used for intervention (e.g. onefinger pushing, rolling, kneading, Circular-rubbing, to-and-fro rubbing, pushing, wiping, cleaning and dissipating, palmtwisting, vibrating, shaking, point-prssing, suppressing, pinching, grasping, finger-twisting, plucking, patting, hitting, flipping, rotating, pulling-extending, back carrying, pulling).	*① In this arm of the study, the tuina therapist will administer a four-step protocol……Step one: relaxation manipulation: The therapist will use his forearm to gently roll on the low back area from the bilateral erector spinae muscles to both thighs, and then continuously from the low back to the gastrocnemius muscle through to the buttocks, for a total of five minutes……Step two: local pressing pain point manipulation: the therapist will apply muscle pressing, stripping, and deep tissue kneading to the pressing pain point in the lumbar region in a direction perpendicular to the erector spinae……Step three: lumbar structural rectification: the therapist will push down (toward the table) and stretch the patient's shoulder anteriorly while stretching the hips posteriorly, rotating the lumbar vertebra along the spinal axis fixing points instantly……Step four: tapping manipulation: the therapist use his or her palm to tap the lumbosacral area for two minutes to generate a warm sensation in deep tissue……*
	Q9. Whether any responses sought of patients during/after massage was reported?	It is considered "yes" if any information about the patient's feel/response for the massage treatment was provided, such as warm feeling, skin reddening, sore and pain, etc.	*① The amount of force used is determined by the patient's Deqi sensation, often described as a dull pain, heaviness, numbness, or soreness, and commonly regarded as an indicator of manipulation effectiveness in acupuncture and tuina..*<br>*②The majority of participants felt comforted by the foot massage as indicated by their positive comments about it, or appeared*

			relaxed by it, indicated by their falling asleep……
	Q10. Whether any measures or management for possible adverse events was pre-mentioned?	It is considered "yes" if any methods (planned or actual implemented) for adverse event prevention and management were provided. If no adverse effect is reported (not including the reporting of no AE identified), it should be scored as "0".	*① Therapeutic safety will be monitored by assessment of patient symptoms as well as blood, urine, and stool tests conducted pre- and post-treatment. Adverse events such as changes in pain, syncope, vertigo, and lumbar function degradation, will be carefully recorded in the case report form.*<br>*②In addition, we evaluated the safety of the interventions. Adverse events associated with the therapy (adverse reactions) and all serious adverse events were recorded at each visit by the therapist. The data were collected by paper-based questionnaires……*
Treatment regimen	Q11. Whether the number, frequency, duration and/or force of provided massage sessions was reported?	It is considered "yes" if the sessions arrangement was provided in detail. If only the force information was absent, it could be scored as "1" when good reporting in other details.	*① Each session lasted for 10 minutes and the sessions were held five times a week, Monday to Friday, from 1:00 pm to 4:00 pm, for a period of 3 weeks. All participants had the opportunity to receive 15 sessions each for the foot massage (FM) intervention and the quiet presence (QP) control.*<br>*② Participants in the two experimental groups then received a 30-min aromatherapy massage or aromatherapy inhalation twice per week for 8 weeks (total of 16 interventions).*
Other components of treatment	Q12. For complex interventions, whether the details of other interventions administered to the massage group were reported?	It is considered "yes" if 1) for complex interventions, details of other treatments administered to the massage; or 2) for single massage interventions, the "not applicable" should be scored as "1". If complex interventions used, relevant RCT guidelines (e.g., cupping, acupuncture, Chinese herbal medicine) should be used to assess the details reporting.	*The comprehensive therapy of acupuncture-moxibustion and Chinese Tuina was adopted, with acupuncture applied first, followed by Chinese Tuina, once daily. a) The acupuncture-moxibustion method: The points selected were Shenmen (HT 7), Sanyinjiao (SP 6), Yinlingquan (SP 9), Xinshu (BL 15), Jueyinshu (BL 14), and Pishu (BL 20). For Sanyinjiao (SP 6) and Yinlingquan (SP 9), the 2 cun filiform needles were inserted perpendicularly 1-1.5 cun deep; Shenmen (HT 7) was punctured with one cun filiform*

			needle inserted perpendicularly 0.3-0.5 cun deep; and Xinshu (BL 15), Jueyinshu (BL 14) and Pishu (BL 20) were punctured with the 1.5 cun filiform needles obliquely inserted 0.5-0.8 cun deep. The rotating reinforcing-reducing method was used. For all the points, after the arrival of qi, the needles were retained for 30 min., during which the needles were manipulated once. Moxibustion was added for points Xinshu (BL 15) and Pishu (BL 20). The moxibustion was operated like this: upon the arrival of qi, a 2-cm moxa stick was put on the needle handle and burned. When the burning of moxa stick finished, the ash was cleaned and the needle taken out. The acupuncture-moxibustion treatment was given once daily, 6 days constituting one therapeutic course, with a 2-day interval between courses. After 3 courses of the treatment, the therapeutic effects were evaluated…….
	Q13. Whether any instruction and/or information of selected massage was presented to treatment providers and the participants?	It is considered “yes” if the article reported any information about the research handbook, investigator brochures, trial notes, booklist, and other forms of instruction.	① *In this study, clinical investigators will submit and explain to each participant the following documents: (1) signed informed consent, (2) information module, (3) authorization to use personal sensitive data based on privacy law, (4) information letter that will be delivered to the family physician, (5) the dedicated educational booklet. This booklet must be considered a part of the SMATH® treatment module……The treatment module is associated with a dedicated educational booklet for each patient that provides general and behavioral indications for preventing Low back pain (LBP). In this clinical study, the educational booklet will be delivered to all enrolled participants to help them correct their lifestyle during and after the intervention period. In this dedicated*

			booklet, different sections explain to the participants spine anatomy, LBP, LBP prevention, advantages of physical activity, suggestions for positions for walking, sleeping, and to keep weight under control, and exercises to prevent LBP.<br>② *Participants were told of the usual pricing structure for shiatsu (a specific type of massage) treatments, the reduced price for those who volunteered for the study, and the refund schedule should a participant not complete all four treatments. Prior to their first treatment, participants were asked to read and sign an informed consent form and were given a copy of the signed form to keep.*
Treatment provider background	Q14. Whether any description of treatment providers' background (e.g., qualification and/or experiences in massage) was reported?	It is considered "yes" if the criteria of massage provider were reported, especially for the qualification and/or experiences. If the information was not in detail, it should be scored as "0".	① *All practitioners in this trial are licensed TCM tuina therapists with at least five years clinical experience in the hospital's Tuina Department. Before taking part in this trial, they will be required to complete a 40-hour training course to master the study protocol. When completed, clinicians will be required to pass an examination during which they are asked to recite the protocol verbally and provide a demonstration of each technique.*
Control or comparator interventions	Q15. Whether the rationale for the choice of the control(s) was provided?	It is considered "yes" if the article reported the rationale for the design or selection of control group.	① *To assess if the massage intervention is effective beyond effects of expectation a sham treatment will be given to the subjects in the control group……The choice to use non-TENS (Transcutaneous electrical nerve stimulations) a sham treatment is based on the fact that TENS has some commonalities to massage; TENS is a form of sensory stimulation, although different to massage, and can be applied to the same limbs that are treated in the massage treatment.*<br>② *The study objectives were to observe the clinical efficacy of*

			primary massage of twining manipulation with one finger (PMTMOF) versus conventional tuina manipulation for treating muscular torticollis……Based on conventional tuina manipulation for treating infantile muscular torticollis, the present study used a modified method—primary massage of twining manipulation with one finger (PMTMOF)—which involves a smaller contact area, provides faster frequency and stronger penetrating force, and produces better effects in promoting blood flow, removing blood stasis, promoting tissue regeneration, subduing swelling, and alleviating pain. It can also be used to locally treat the sternocleidomastoid muscle and promote development of adjacent muscle. PMTMOF produced more obvious curative treatment effects on infantile muscular torticollis than conventional tuina manipulation and could effectively shorten treatment time and avoid sequelae due to delayed healing
	Q16. Whether the details of control or comparator were described?	It is considered "yes" if the article reported the details of control group, relevant RCT guidelines should be used to assess the details reporting.	① *Patients in the control group will receive a conventional pharmacological treatment regimen of one 0.3 g capsule of sustained-release ibuprofen, taken three times each day (Ibuprofen Sustained Release Capsules, 0.3 g per capsule, Sino-GlaxoSmithKline, Tianjin, China)*<br>② *The sham device (medical device without active principles) will be set-up with the same treatment variables as the SMATH® device (system for automatic thermomechanic massage in health). The noise and vibration generated by the sham device will be the same as that of the SMATH® system because the devices have the same mechanical configuration, including the same motor and*

			transmission equipment, operating in the same mode. Despite this similarity, a therapeutic placebo effect could be introduced by the sham device. This risk has been considered and accepted as a natural risk of the RCT with a sham control. Participants assigned to the sham therapy arm will receive the same treatment module scheduled for the active SMATH® arm (14 sessions in four weeks from T2 to T4). The participants in the sham arm will also receive the educational booklet. The SMATH® and sham devices are located inside two different therapy boxes and treatment will be given at separate times to prevent contact between participants in the different treatment arms. The operators managing treatments will be the same for the SMATH® and sham devices, and they have been trained to answer the participants' questions about the procedures. The same operators will also be trained and educated in keeping participants in the study, avoiding an increased drop-out rate. The main features of the SMATH® system and sham version are presented in Figures 4 and 5, respectively.
